# Plant‐Derived Extracellular Vesicles: Biogenesis, Function, and Application in Cancer Immunotherapy

**DOI:** 10.1002/ptr.70456

**Published:** 2026-09-21

**Authors:** Guosheng Li, Mengyu Li, Shujing Liu, Yuan Wei, Chen Wang

**Affiliations:** ^1^ School of Pharmacy Jiangsu University Zhenjiang Jiangsu China

**Keywords:** cancer immunotherapy, cross‐kingdom regulation, drug delivery, plant‐derived extracellular vesicles (PDEVs), traditional Chinese medicine (TCM)

## Abstract

Plant‐derived extracellular vesicles (PDEVs) are nanoscale, lipid bilayer‐enclosed vesicles secreted by plant cells. They are widely distributed across plant tissues, including fruits, roots, stems, and leaves, and are particularly abundant in edible and medicinal plant species. This review presents a comprehensive overview of recent advances in PDEV research, focusing on their biogenesis, isolation strategies, physicochemical characterization, and cross‐kingdom regulatory mechanisms. We highlight the multifaceted roles of PDEVs in anti‐tumor immunity, including tumor cell suppression, immune stimulation, drug delivery, and gut‐microbiota‐mediated immunomodulation. Despite their therapeutic potential, challenges remain in standardization and pharmacokinetic profiling. Emerging engineering strategies offer promising solutions to improve targeting and therapeutic efficacy. Additionally, PDEVs provide a unique platform for mechanistic exploration of traditional Chinese medicine. By integrating current knowledge and future perspectives, this review aims to advance the translational application of PDEVs in cancer immunotherapy and dietary intervention.

## Graphical abstract

This illustration shows the biogenesis pathways of edible and medicinal plant‐derived extracellular vesicles (PDEVs) and their therapeutic effects following uptake in the human body, highlighting their potential for application in cancer immunotherapy.

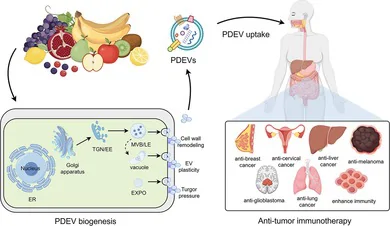

**Abbreviations**
AFM: atomic force microscopy
ANN1: annexin 1
ANN2: annexin 2
APCs: antigen‐presenting cells
BBB: blood–brain barrier
BENs: exosome‐like nanoparticles from broccoli
CDS: coding sequence
CTLs: cytotoxic T lymphocytes
DC: dendritic cell
DGDG: digalactosyldiacylglycerol
DHA: docosahexaenoic acid
DOX: doxorubicin
DUC: differential ultracentrifugation coupled
EE: early endosome
ER: endoplasmic reticulum
ESCRT: endosomal sorting complex required for transport
EXPO: exocyst‐positive organelle
F/B: Firmicutes‐to‐Bacteroidetes
GC–MS: gas chromatography–mass spectrometry
GI: gastrointestinal
GMP: Good Manufacturing Practice
HSP70: heat shock protein 70
i.p.: intraperitoneal
i.v.: intravenous
IAC: immunoaffinity capture
IBD: inflammatory bowel disease
ICG: indocyanine green
ILVs: intraluminal vesicles
LC–MS: liquid chromatography‐mass spectrometry
LE: late endosome
MGDG: monogalactosyldiacylglycerol
MS: mass spectrometry
MVBs: multivesicular bodies
NKTs: natural killer T cells
NSCLC: Non‐small cell lung cancer
NTA: nanoparticle tracking analysis
OVA: ovalbumin
PA: phosphatidic acid
PATL1: patellin1
PC: phosphatidylcholine
PDEVs: plant‐derived extracellular vesicles
PE: phosphatidylethanolamine
PEG: polyethylene glycol
PEN1: penetration 1
PEN3: penetration 3
PI: phosphatidylinositol
PLC: phospholipase C
PS: phosphatidylserine
PTX: paclitaxel
qPCR: quantitative polymerase chain reaction
RIN4: RPM1‐interacting protein 4
RISC: RNA‐induced silencing complex
ROS: reactive oxygen species
s.c.: subcutaneous
SEC: size‐exclusion chromatography
siRNA: small interfering RNA
SOPs: standard operating procedures
TAM: tumor‐associated macrophages
TCM: traditional Chinese medicine
TEM: transmission electron microscopy
TET8: tetraspanin8
TGN: trans‐Golgi network
TME: tumor microenvironment
UC: ultracentrifugation
UF: ultrafiltration
WB: western blotting

## 1 Introduction

Edible and medicinal plants exemplify the dual nutritional and therapeutic value of many foods, offering preventive and curative effects against diseases (Debri et al. 2026). This concept aligns with the traditional Chinese medicine (TCM) principle of “prevention before disease onset,” which emphasizes maintaining the balance of “yin” and “yang” and strengthening the body's “zheng qi” through dietary practice (Wang 2005). Plant‐derived extracellular vesicles (PDEVs) have emerged as mediator of cross‐kingdom communication, offering a promising strategy for the mechanistic investigation of TCM. PDEVs are nanoscale vesicles naturally secreted by plant cells, characterized by a phospholipid bilayer membrane and the ability to encapsulate a wide range of bioactive molecules, including proteins, lipids, RNA, and metabolites (Figure 1). Owing to their favorable biocompatibility, low toxicity, ease of isolation, and diverse biological activities, PDEVs have attracted considerable attention as novel functional materials and drug delivery carriers (Han et al. 2025). They also demonstrate potential as modernized formulations for TCM. Research on PDEVs dates back to the last century. In 1967, Halperin and Jensen first observed multivesicular body (MVB) structures in carrot cell cultures by transmission electron microscopy (TEM) (Halperin and Jensen 1967). In 2009, Regente et al. isolated vesicles with characteristic phospholipid membrane structures from the extracellular fluid of sunflower seeds (Regente et al. 2009). This study explored the role of PDEV in intercellular communication and provided key biochemical evidence. PDEVs have since attracted growing interest within the scientific community. In 2013, the Nobel Prize in Physiology or Medicine was awarded to scientists who made significant contributions to the understanding of regulatory mechanisms governing intracellular vesicle transport (Fischer von Mollard et al. 1990; Novick et al. 1980; Söllner, Bennett, et al. 1993; Söllner, Whiteheart, et al. 1993). This recognition accelerated research on exosomes and provided valuable theoretical and technical foundations for the study of PDEVs. In 2018, Cai et al. reported that plants can deliver small RNAs (sRNAs) to pathogenic fungi via extracellular vesicles (EVs), thereby enabling cross‐kingdom RNA interference (Cai et al. 2018). This groundbreaking finding demonstrated the regulatory capacity of PDEVs across kingdoms and established a new paradigm for understanding plant–pathogen interactions, greatly increasing research interest in this field.

**FIGURE 1:**
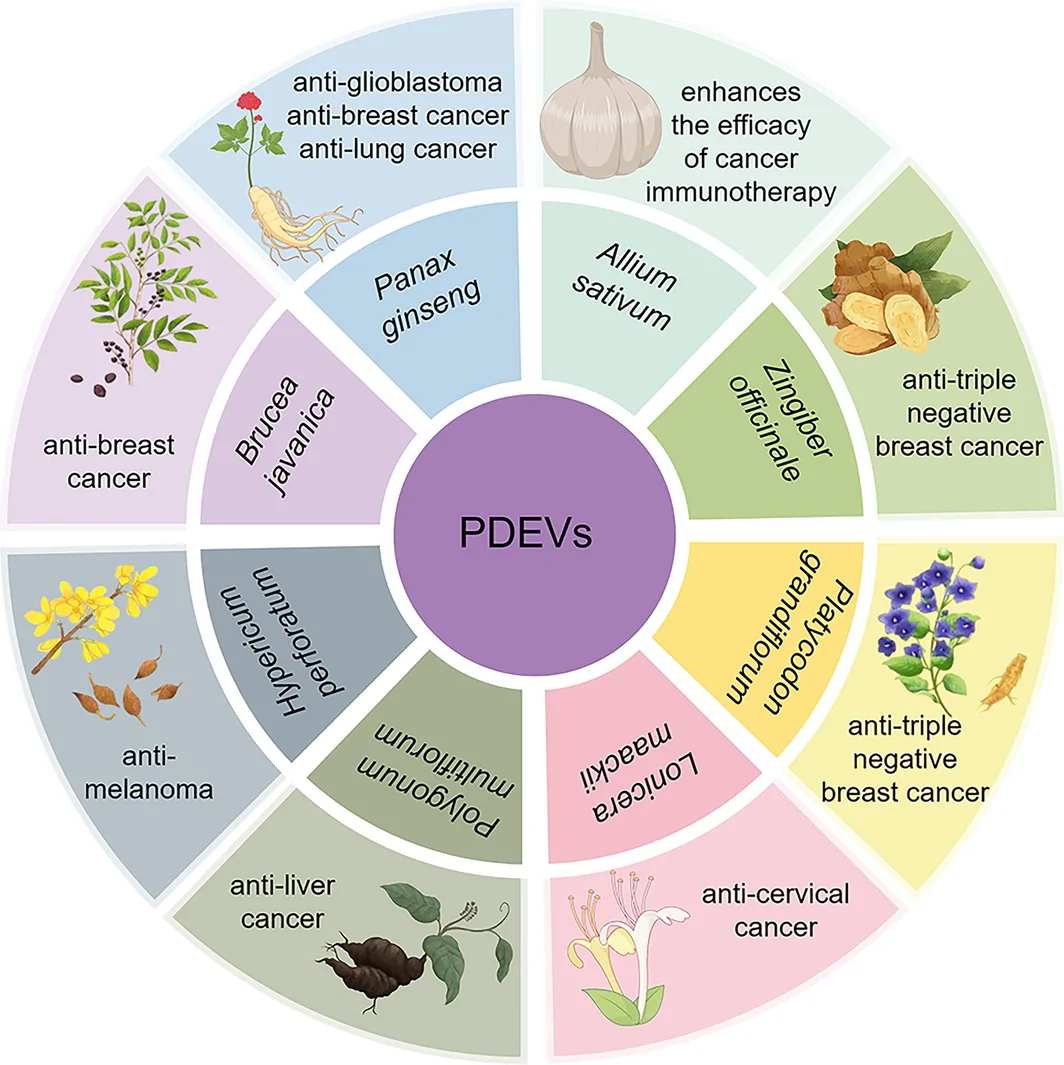
Anti‐tumor effects of PDEVs derived from edible and medicinal plants.

Cancer immunology focuses on the characteristics of cancer antigens, the interactions between the immune system and tumor development, and the associated diagnostic and therapeutic strategies. Its core mechanisms involve both humoral and cellular immunity, with T cells, natural killer T cells (NKTs), and macrophage‐mediated immune responses playing pivotal roles in anti‐cancer immunity. However, cancer cells can evade immune surveillance through multiple mechanisms (Myers and Miller 2021). Recent studies indicate that PDEVs enhance T cell function by delivering bioactive molecules while simultaneously suppressing mechanisms of tumor immune escape (Zhu et al. 2025), highlighting their potential as a promising platform for cancer immunotherapy.

This review provides a comprehensive update on the biology of PDEVs, their role in cross‐kingdom regulation mediated by molecules such as microRNAs (miRNAs), and their applications in cancer therapy, with particular emphasis on immune regulation. We also explore emerging directions, including PDEV engineering and TCM‐informed mechanistic studies, aiming to bridge fundamental research with clinical translation.

## 2 PDEVs

### 2.1 Biological Characteristics of PDEVs

PDEVs mediate intercellular communication and material transport. Three biogenesis pathways of PDEVs have been identified: the MVB pathway, the exocyst‐positive organelle (EXPO) pathway, and the vacuolar pathway (Cong et al. 2022; Hu et al. 2020). The classical MVB pathway begins with endosome formation, during which intracellular cargo such as proteins, nucleic acids, and lipids is selectively packaged into intraluminal vesicles (ILVs) through inward budding of the endosomal membrane. This process is mainly mediated by the endosomal sorting complex required for transport (ESCRT) machinery and associated regulatory systems. Upon maturation, MVBs fuse with the plasma membrane, releasing ILVs into the extracellular space (Ambrosone et al. 2023). The EXPO pathway involves direct budding from the plasma membrane in a manner independent of the classical endosomal sorting system (Gao et al. 2026). The vacuolar pathway proposes that fusion of vacuoles with the plasma membrane may release vesicle populations distinct from those derived from MVBs (Hu et al. 2020). The relative physiological relevance of these pathways, their potential coexistence across different cell types or physiological conditions, and the specific molecular machinery governing each route remain active areas of investigation.

Different PDEV biogenesis pathways produce vesicle populations with partially overlapping but distinct size distributions, although heterogeneity is also observed within each category. MVB‐derived vesicles (exosome‐like) typically range from 30 to 200 nm in diameter. Their relatively uniform size reflects the regulated nature of ESCRT‐dependent or ESCRT‐independent formation within endosomal compartments (Mu et al. 2023). EXPO‐derived vesicles generally exhibit larger and more variable sizes, typically ranging from 200 to 500 nm. Their size heterogeneity may reflect differences in membrane curvature regulation and cargo composition (Gao et al. 2026). Vacuolar pathway–derived vesicles show the broadest size distribution, ranging from approximately 50 to 500 nm, encompassing both small vesicles and larger populations depending on vacuolar content and fusion dynamics.

A key distinction in PDEV release is that, in plant cells, vesicle secretion must overcome the rigid cell wall—a structural barrier absent in animal cells. Several mechanisms have been proposed to explain how PDEVs traverse this barrier. One is enzymatic remodeling, where cell wall–degrading enzymes such as pectinases or cellulases, either associated with PDEVs or secreted by plant cells, locally loosen or modify the cell wall architecture, thereby facilitating vesicle passage (Woith et al. 2021). Another proposed mechanism is physical deformation, in which vesicles exploit their structural flexibility to pass through cell wall pores smaller than their diameter by transiently altering their shape, for example, from spherical to tubular forms. A third proposed mechanism involves mechanical forces, such as turgor pressure, which may drive EVs through the cell wall (Rutter and Innes 2018). In addition, enhanced vesicle diffusion may occur during pathogen attack or under specific physiological conditions, driven by changes in cell wall structure and permeability (Movahed et al. 2019).

Following secretion into the extracellular space, PDEVs mainly exert their biological functions through internalization by recipient (target) cells. The main mechanisms underlying this uptake include endocytosis (Ju et al. 2013) and receptor‐mediated signaling (Song et al. 2020). Among these, endocytosis is the most extensively characterized and widely accepted pathway (Figure 2).

**FIGURE 2:**
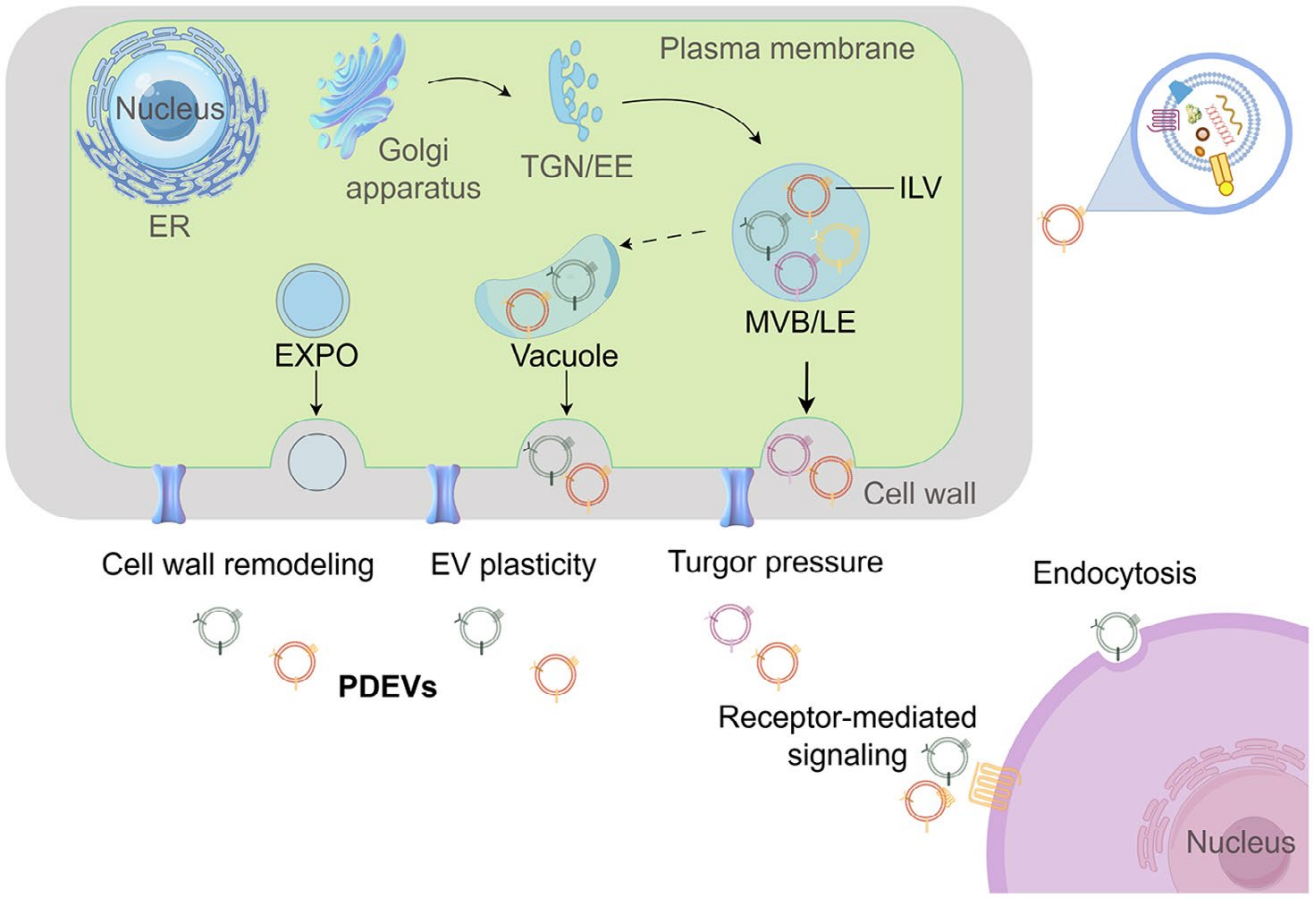
Biogenesis and uptake of PDEVs. PDEVs are generated via the MVB pathway, the EXPO pathway, and the vacuolar pathway. Their release across the rigid plant cell wall is facilitated by mechanisms such as cell wall remodeling, vesicle plasticity, and turgor pressure. Subsequently, recipient mammalian cells internalize these vesicles through endocytosis and receptor‐mediated signaling. Abbreviations: EE, early endosome; ER, endoplasmic reticulum; EXPO, exocyst‐positive organelle; ILV, intraluminal vesicle; LE, late endosome; MVB, multivesicular body; TGN, trans‐Golgi network.

The diverse functions of PDEVs are mainly attributed to their cargo of bioactive molecules. These molecules are selectively packaged during endosomal vesicle formation. Both ESCRT machinery and non‐ESCRT pathways (such as those associated with lipid raft microdomains) contribute to the encapsulation of diverse cargo, including proteins, nucleic acids, and lipids (Gao et al. 2014). Among these components, miRNAs delivered to recipient cells via PDEVs serve as key regulatory molecules. MiRNAs are non‐coding, single‐stranded RNAs of approximately 20–24 nucleotides that typically bind to complementary sequences in the 3′ untranslated regions of target messenger RNAs. This interaction inhibits mRNA translation or promotes mRNA degradation (Carthew and Sontheimer 2009). The lipid bilayer of PDEVs protects encapsulated miRNAs from nuclease degradation, thereby preserving their stability and functional integrity during long‐distance transport in biological fluids.

### 2.2 Preparation Methods of PDEVs

Based on the differences in isolation methods, PDEVs can be classified into two categories (Janouskova et al. 2022). The first category consists of naturally secreted vesicles, obtained from supernatants of plant tissue cell culture (Zhu et al. 2025), or through vacuum infiltration of plant tissues, mainly leaves (Liu, Wu, et al. 2020). These vesicles are purified using differential centrifugation, ultracentrifugation, and density gradient centrifugation. They exhibit high purity and are suitable for studies investigating the physiological roles of PDEVs in plants, such as their involvement in resistance to external infections (Cai et al. 2018; Wang, He, et al. 2024). The second category comprises nanovesicles isolated from plant roots, leaves, and fruits using disruptive methods such as grinding (Lee et al. 2020), extruding (You et al. 2021), or juicing (Cao et al. 2019). Although these vesicles are purified using procedures similar to those applied to the first category, it remains uncertain whether they exist naturally in plants.

In general, the isolation and enrichment of PDEVs involve several steps.

The first step is rigorous sample pretreatment to obtain a crude extract while preserving vesicle integrity. The choice of method depends on the plant species and tissue type. For tough tissues such as fresh rhizomes (e.g., ginger), homogenization is used, whereby plant cells are mechanically disrupted to release vesicles into a pre‐chilled extraction buffer. This is followed by the removal of fibers and starch through differential centrifugation (Kim et al. 2022; Li, Wang, et al. 2024; Seo et al. 2023). For leaf samples, pretreatment typically involves chopping, followed by soaking or low‐speed homogenization (Jokhio et al. 2024; Lo et al. 2025). An alternative technical approach involves the purification of naturally secreted vesicles from apoplastic fluid (Chen, He, and Jin 2022; Eldahshoury et al. 2024; Yang et al. 2020). For succulent fruits such as citrus or grapes, juice obtained using a juicer is subjected to mild enzymatic digestion with pectinase and cellulase to reduce viscosity (Biel‐Nielsen et al. 2025; Cui, Liu, et al. 2024; Rahimi Ghiasi et al. 2018). All procedures should be conducted on ice to minimize thermal damage and preserve vesicle integrity. Homogenization buffers typically contain osmotic stabilizers (e.g., sucrose), pH buffers (such as HEPES), and protease inhibitors to maintain vesicle stability and prevent degradation of intravesicular contents.

Afterward, sequential centrifugation is performed to remove impurities from the crude extract, including cell debris and large particulate contaminants. Coarse filtration is first conducted by passing the extract through multiple layers (e.g., 2–4) of sterile gauze or nylon mesh, thereby removing most tissue remnants and collecting the resulting filtrate. The filtrate is then subjected to low‐speed centrifugation (3000–10,000× *g*, 15–30 min, 4°C) to separate the PDEV‐containing supernatant (which also contains soluble proteins and organelles) from the pellet, which consists of intact cells, nuclei, chloroplasts, and starch granules (Kilasoniya et al. 2023; Zhang, Viennois, et al. 2016). The supernatant is then carefully collected by aspiration.

Subsequently, depth filtration is performed for further clarification. To remove residual subcellular debris and large protein aggregates, the collected supernatant is passed through sterile membrane filters with pore sizes of 0.45 or 0.22 μm (Huang et al. 2021; Wang et al. 2014). Filtration at 0.22 μm effectively eliminates bacteria while producing a highly clarified solution, thereby providing a foundation for subsequent high‐resolution purification steps such as ultracentrifugation (UC) or size‐exclusion chromatography (SEC). Notably, depth filtration may lead to partial loss of PDEVs due to adsorption or retention; therefore, its necessity should be carefully considered based on specific research objectives (Dragovic et al. 2015).

The final purification methods for PDEVs involve several approaches, each with distinct principles and performance characteristics that depend on sample type, experimental conditions, and specific research objectives. The choice of method requires careful consideration of the trade‐offs between yield, purity, structural integrity, and compatibility with downstream applications. These approaches include centrifugation‐based methods: UC (Zhang, Viennois, et al. 2016) and density gradient centrifugation; size‐based separation techniques such as ultrafiltration (UF) (Liu, Wu, et al. 2020) and SEC (Berger et al. 2020); affinity‐based approaches including immunoaffinity capture (IAC) (He et al. 2021; Huang et al. 2021); precipitation‐based methods such as polyethylene glycol (PEG)‐based precipitation (Kalarikkal et al. 2020).

#### 2.2.1 Integrated Purification Strategies for PDEVs

To overcome the limitations of single‐step separation methods and achieve the level of purity required for specific applications, the integration of multiple separation techniques has become a widely adopted strategy. By combining complementary methods, synergistic workflows can significantly improve both isolation yield and purity. Commonly used optimization approaches include:

UC‐SEC: This approach first employs UC for rapid concentration and preliminary purification, followed by SEC to remove small‐molecule impurities such as co‐precipitated proteins and residual contaminants. This combination effectively enhances vesicle purity while preserving structural integrity and has been successfully applied to isolate EVs from *Brassica oleracea* (cabbage) cells (You et al. 2021).

UC combined with polymer‐based precipitation: This method typically involves initial enrichment using commercial polymer precipitation kits, followed by UC to remove residual polymers and further purify the sample. This approach offers a favorable balance between efficiency and cost‐effectiveness for applications where moderate purity is acceptable and has been used in the extraction of EVs from *Panax ginseng* (Zhu et al. 2025).

Density gradient centrifugation combined with UC: As noted above, this combination currently represents the method of choice for high‐purity EV isolation in basic research, particularly when structural preservation and the removal of non‐vesicular contaminants are critical for downstream functional studies (Chen, He, and Jin 2022; Pascucci and Scattini 2021; Szatanek et al. 2017; Tiwari et al. 2021; Zhou et al. 2024).

### 2.3 Characterization of PDEVs

The characterization of PDEVs requires a combination of complementary techniques to comprehensively evaluate their physicochemical properties, biochemical composition, and morphological features (Table 1). Commonly employed analytical approaches include nanoparticle tracking analysis (NTA), microscopy, protein profiling, nucleic acid analysis, lipidomics, and metabolomics.

**TABLE 1:** Methods for characterization of PDEVs.

Analysis	Technique	Display
Nanoparticle tracking analysis (NTA)	Brownian motion of individual particles	Size distribution and particle concentration
Microscopy	Electromagnetic lens imaging; interatomic forces	Morphology, lipid bilayer structure, particle size; surface roughness
Protein analysis	Antibody–Antigen interaction	Transmembrane‐/membrane‐associated proteins (PEN1 and TET8)
Nucleic acid analysis	High‐throughput sequencing, qPCR	miRNAs and siRNAs (miR2911, miR168a, etc.)
Lipidomics	MS	Lipid profiles (percentage of each lipid)
Metabolomics	MS, LC–MS, GC–MS	Secondary metabolites (e.g., flavonoids and phenols)

NTA employs a laser beam to illuminate the sample and uses a camera to track the Brownian motion of individual particles in real time, enabling the calculation of hydrodynamic diameter and particle concentration based on the Stokes‐Einstein equation (Vestuto et al. 2024). Microscopy techniques include TEM and atomic force microscopy (AFM). TEM enables direct visualization of vesicle ultrastructure (Tiwari et al. 2021), while AFM scans the sample surface with a probe mounted on a microcantilever, detecting interaction forces between the tip and the surface to reconstruct three‐dimensional topographical images (Szatanek et al. 2017).

Protein analysis is performed via Western blotting and proteomics, in which proteins are separated and detected to identify characteristic PDEV protein markers. In contrast to the well‐characterized tetraspanin family of membrane proteins in mammalian exosomes (e.g., CD63 and CD81), definitive marker proteins for PDEVs remain incompletely defined. Nevertheless, several plant‐specific or enriched proteins, such as penetration 1 (PEN1) and tetraspanin 8 (TET8), have been identified as candidate markers (Koch et al. 2025; Liu, Wang, et al. 2020). In addition, RPM1‐interacting protein 4 (RIN4), patellin 1 (PATL1), penetration 3 (PEN3), and annexin 1 (ANN1) have been experimentally identified as potential markers based on their resistance to protease digestion in the absence of detergent (Koch et al. 2025). Notably, TET8 and PEN1 represent distinct subpopulations of plant EVs, with colocalization observed in only 4.7% of vesicles. PEN1, PATL1, and ANN2 commonly mark the same EV population, which is distinct from TET8‐positive EVs, whereas RIN4 is more frequently associated with TET8‐positive EVs (Ghosh and Innes 2025).

PDEVs are enriched in small RNAs, particularly miRNAs and siRNAs, which are considered key mediators of cross‐kingdom regulatory functions and serve as important biomarkers for PDEV characterization. Nucleic acid analysis involves the extraction of total nucleic acids from PDEVs, followed by high‐throughput sequencing, quantitative polymerase chain reaction (qPCR), or related techniques (Cai et al. 2018; Chi et al. 2024; Zhao et al. 2025). Lipid components are closely associated with PDEV biogenesis, structural stability, and biological function. Lipidomics mainly relies on mass spectrometry (MS) to systematically characterize the lipid composition of PDEV membranes (Wang, Li, et al. 2025). Metabolomics analyses are performed using MS, liquid chromatography–mass spectrometry (LC–MS), and gas chromatography–mass spectrometry (GC–MS) (Kaimuangpak, Srisongkram, et al. 2024).

The isolation and characterization procedures of PDEVs are illustrated in Figure 3.

**FIGURE 3:**
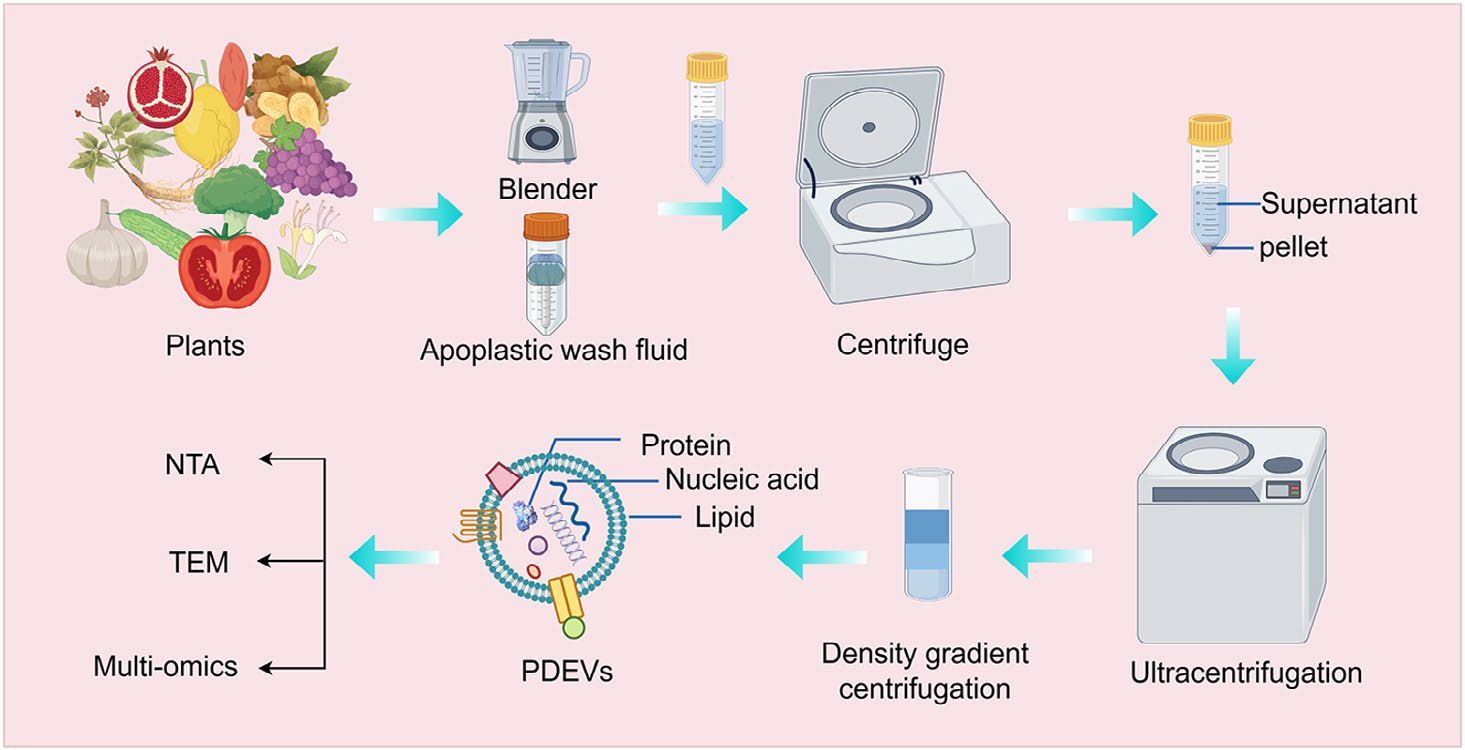
Isolation and characterization of PDEVs. NTA, nanoparticle tracking analysis. TEM, transmission electron microscopy. Multi‐omics include proteomics, nucleic acid analysis, lipidomics, and metabolomics.

Critical considerations in PDEV characterization: distinguishing vesicles from non‐vesicular components. A major challenge in PDEV research is the reliable discrimination of bona fide vesicles from co‐isolated non‐vesicular nanoparticles, aggregates, and plant‐derived contaminants. The reported size range of 50–500 nm includes a heterogeneous mixture of particle types commonly present in plant‐derived preparations, such as true extracellular vesicles (typically 50–200 nm for exosome‐like vesicles, with some populations extending to 200–500 nm), non‐vesicular nanoparticles including lipid droplets, protein aggregates, and ribonucleoprotein complexes, plant‐derived contaminants such as starch granules, cell wall fragments, and aggregated secondary metabolites, as well as artifacts that may arise during tissue disruption and homogenization (Huang et al. 2025). To address this complexity, multiple characterization strategies should be employed. Morphological validation: TEM combined with immunogold labeling using antibodies against established plant vesicle markers (e.g., PEN1 and TET8) provides direct evidence of vesicular identity and helps distinguish vesicles from amorphous aggregates. Cryo‐electron microscopy offers superior preservation of native structure and can reveal characteristic lipid bilayer morphology (Chaya et al. 2024). Buoyant density analysis: True vesicles typically float at defined densities in density gradients (e.g., 1.04–1.12 g/mL) (Koch et al. 2025), whereas co‐isolated contaminants often exhibit different buoyant densities and can be separated under appropriate gradient conditions (Bokka et al. 2020). Proteomic profiling: Identification of vesicle‐associated proteins (e.g., ESCRT components, tetraspanin‐like proteins, and membrane trafficking proteins), together with the absence of abundant cytoplasmic contaminants such as Rubisco, strengthens evidence for vesicle identity (Rodríguez de Lope et al. 2025). Size distribution analysis: NTA results should be interpreted with caution, as particle counts include all suspended nanoparticles that fall within the instrument detection range. Therefore, integration with orthogonal techniques (e.g., tunable resistive pulse sensing and flow cytometry), along with comparison to microscopy‐based size measurements, is essential for reliable interpretation. Reported concentration values should be regarded as operational estimates dependent on specific isolation protocols rather than absolute vesicle counts (Vestad et al. 2017).

### 2.4 PDEVs Versus Mammalian Exosomes

PDEVs and mammalian exosomes differ significantly in biogenesis, composition, and function, giving PDEVs distinct advantages for various applications (Mu et al. 2023). They are widely available and sustainable, as they are abundant in plant roots, stems, leaves, fruits, and sap. Importantly, their raw materials can be sourced at scale from agricultural byproducts (such as fruit peels and straw) or other low‐cost plant biomass. This ensures cost‐effectiveness while also aligning with green and sustainable production principles (Jung et al. 2025). The following sections outline the advantages of PDEVs over mammalian exosomes (Figure 4).

**FIGURE 4:**
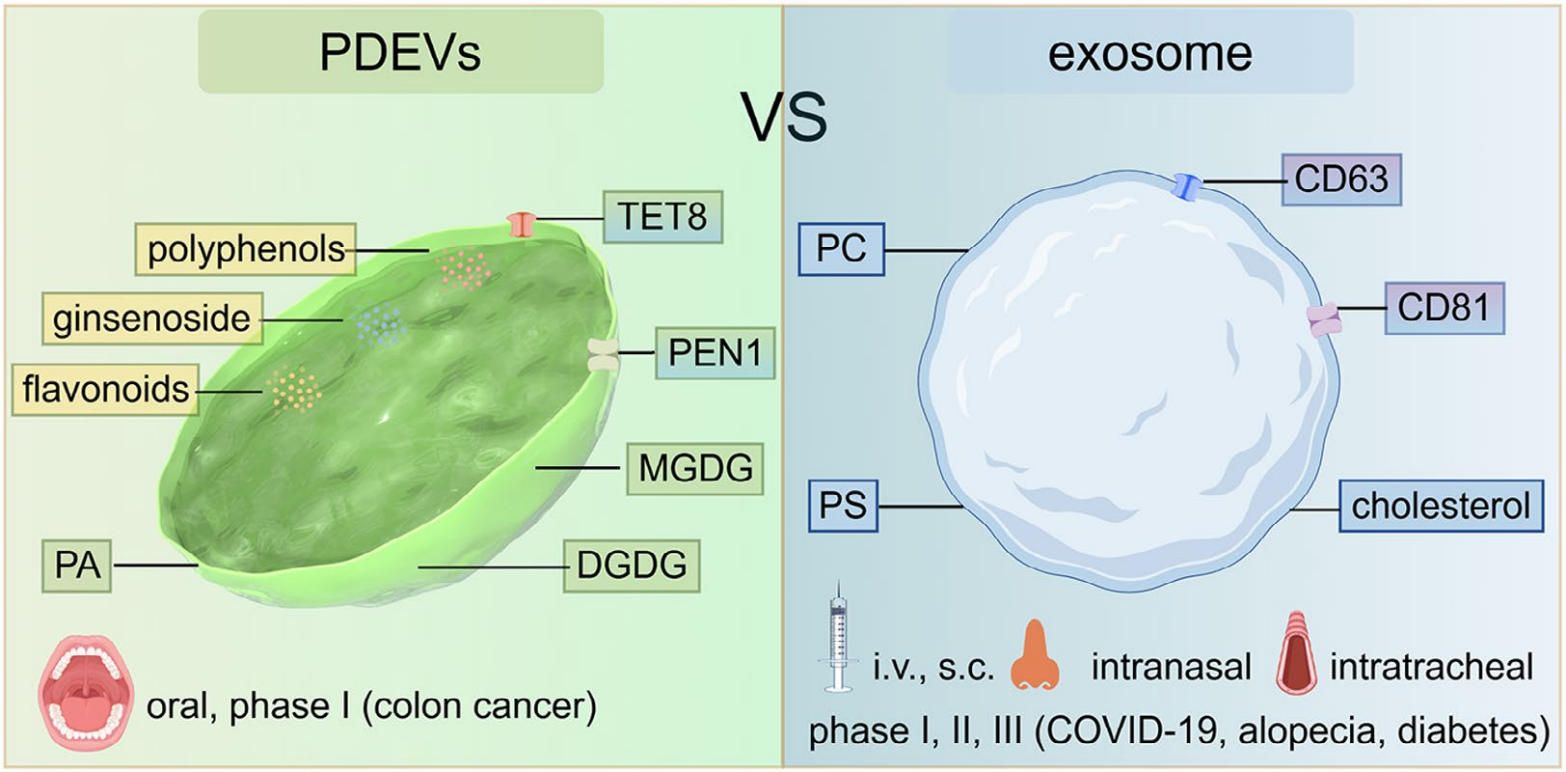
Differences between PDEVs and mammalian exosomes in composition and translation potential. Surface proteins: TET8, PEN1; CD63, CD81. *Surface lipids*: PA (phosphatidic acid), DGDG (digalactosyldiacylglycerol), MGDG (monogalactosyldiacylglycerol); PC (phosphatidylcholine), PS (phosphatidylserine), cholesterol. *Metabolite cargo*: Flavonoids, polyphenols, ginsenoside. *Interventional study*: Phase I, II, III clinical trial. *Administration routes*: Oral; i.v. (intravenous), s.c. (subcutaneous), intranasal, intratracheal.

#### 2.4.1 Unique and Diverse Bioactive Components

PDEVs typically contain not only common proteins and nucleic acids but also plant‐specific secondary metabolites such as flavonoids and phenolic acids (López de Las Hazas et al. 2023). These characteristic components may serve as the molecular basis for their distinct pharmacological activities, underpinning their functional uniqueness. Ginger‐derived EVs are enriched in gingerols and shogaols, which contribute to their anti‐inflammatory and intestinal protective effects (Zhang, Viennois, et al. 2016). Grapefruit‐derived EVs contain high levels of naringin and naringenin, which are associated with anti‐tumor and immunomodulatory activities (Wang et al. 2014).

#### 2.4.2 Biosafety Considerations

EVs derived from commonly consumed edible plants such as grapes, ginger, and lemon generally demonstrate favorable biocompatibility and low immunogenicity in preclinical models under defined experimental conditions. For example, in vitro studies have reported that grape‐derived EVs show no significant cytotoxicity in human intestinal cell lines at concentrations up to 100 μg/mL (Ju et al. 2013). Similarly, in vivo studies indicate that oral administration of ginger‐derived EVs at doses up to 20 mg/kg in mice does not produce observable adverse effects or systemic inflammatory responses (Zhang, Viennois, et al. 2016). However, these safety profiles are highly context‐dependent and cannot be generalized across all plant species, vesicle preparations, or administration routes, although oral delivery remains the most widely studied and favorable approach. Notably, certain preparations (e.g., tea‐derived PDEVs (Chen et al. 2023)) may induce hepatorenal toxicity following intravenous administration. The immunogenic and allergenic potential of PDEVs is influenced by multiple factors, including plant source, isolation method, purity, dosing regimen, and recipient characteristics. Moreover, robust methodologies are urgently needed to elucidate the metabolic fate of PDEVs across different routes of administration. To date, available evidence is derived almost exclusively from preclinical models (e.g., mice), with no reported clinical trial data. A comprehensive evaluation of the long‐term safety profile and potential immunogenicity of PDEVs following repeated administration in humans remains to be conducted. Systematic pharmacotoxicity assessments, together with in‐depth characterization of immune responses, are essential prerequisites for clinical translation (Lopez‐Rodriguez et al. 2019; Mu et al. 2023).

#### 2.4.3 Gastrointestinal Stability

PDEVs exhibit great stability in the gastrointestinal (GI) tract, mainly due to their natural lipid bilayer membrane, which protects encapsulated bioactive molecules from degradation by gastric acid, proteolytic enzymes, and bile salts (Ning et al. 2025; Zuo et al. 2025). For example, *Dioscorea polystachya* (yam)‐derived EVs maintain structural integrity in simulated GI fluids (Yao et al. 2026). Ginger‐derived EVs have been used as delivery vehicles for TNF‐α siRNA, demonstrating strong resistance to gastric acid degradation (Cui, Du, et al. 2024). Similarly, exosome‐like nanoparticles from broccoli (BENs) exhibit high GI stability (Duan et al. 2023).

### 2.5 Mechanisms of Cross‐Kingdom Regulation Mediated by PDEVs

PDEVs not only mediate intercellular communication within their host plants but also exhibit cross‐kingdom regulatory capabilities (Ren et al. 2026). Evidence suggests that orally administered PDEVs may modulate mammalian gut microbiota homeostasis and host immune responses, thereby exerting anti‐inflammatory (Yi et al. 2023), antioxidant (Batsukh et al. 2024), anti‐tumor (Chu et al. 2024; Cong et al. 2022; Karamanidou and Tsouknidas 2021; Yi et al. 2025) effects.

One core function of EVs is to serve as natural carriers of nucleic acids. PDEVs can transfer functional genetic material, such as mRNA and miRNA, to recipient cells, enabling the regulation of gene expression in foreign cellular environments and facilitating the exchange of genetic information from intercellular to cross‐species levels (Liu et al. 2021; Olmi et al. 2023; Valadi et al. 2007).

#### 2.5.1 Targeting Fungal Pathogens

In plant–fungal interactions, the cross‐kingdom regulatory mechanisms of PDEVs are relatively well defined. For example, *Arabidopsis thaliana* ‐derived EVs can deliver host small RNAs into *Botrytis cinerea* cells. These sRNAs (e.g., TAS1c‐siR483 and TAS2‐siR453) bind to mRNAs of pathogen virulence‐associated genes through near‐perfect complementary base pairing, leading to mRNA cleavage and degradation via the RNA‐induced silencing complex (RISC), thereby effectively suppressing fungal pathogenicity (Cai et al. 2018). This mechanism relies on high sequence specificity.

#### 2.5.2 Action in Mammalian Systems

The regulatory effects of PDEVs on mammals are complex and remain an active area of research. Evidence suggests that plant miRNAs may have potential applications in disease diagnosis and treatment. For example, miR156a derived from broccoli has been reported to inhibit metastasis in nasopharyngeal carcinoma cells, potentially through regulation of metastasis‐associated genes (Tian et al. 2016), while miR168a has been implicated in regulating liver function in mice (Zhang et al. 2012). Current evidence indicates that plant miRNAs may function similarly to endogenous animal miRNAs by binding to target mRNAs within coding sequences (CDS) (Zhang et al. 2012) or 3′ untranslated regions (3′‐UTR) (Hou et al. 2018) via sequence complementarity, leading to translational repression. Target downregulation appears to depend on the degree of base pairing: extensive complementarity favors mRNA degradation (Hou et al. 2018), whereas partial complementarity typically results in translational inhibition without substantial changes in transcript abundance (Zhang et al. 2012). Nevertheless, this field remains controversial, and further experimental evidence is needed to clarify the stability, effective concentrations, and precise mechanisms of plant miRNAs in mammalian systems.

GI stability is a major bottleneck in the oral delivery of biomacromolecules such as miRNAs and proteins. PDEVs have emerged as a promising strategy due to their innate lipid bilayer membrane, which effectively protects therapeutic cargo from degradation. In contrast to mammalian exosomes, whose membranes are protein‐rich, the diverse lipid composition of PDEV membranes contributes to their enhanced stability and lower immunogenicity (Zheng et al. 2026). Critically, the lipid profile of PDEVs differs fundamentally from that of mammalian exosomes.

The lipid composition of PDEVs (Table 2) is predominantly characterized by phosphatidic acid (PA), digalactosyldiacylglycerol (DGDG), and monogalactosyldiacylglycerol (MGDG) (Huang et al. 2025). These plant‐specific lipids confer enhanced protection to PDEVs against the harsh gastrointestinal environment, including gastric acid in the stomach and bile salts in the intestine, with changes in size and surface charge observed under simulated gastric (pH 2.0) and intestinal (pH 6.5) conditions (Mu et al. 2014; Nemidkanam and Chaichanawongsaroj 2022; Zhang, Viennois, et al. 2016).

**TABLE 2:** Lipid composition of PDEVs and milk‐derived EVs.

Source	Phospholipid (PL)		Glycerolipid (GL)		References
	PA	PC	DGDG	MGDG	
Ginger	25%–40%	/	25%–40%	20%–30%	(Zhang, Viennois, et al. 2016)
	35.2%	/	/	/	(Teng et al. 2018)
	46.6%	/	41.87%	/	(Zheng et al. 2026)
	41.52%	0.10%	33.90%	14.05%	(Teng et al. 2025)
Turmeric	34.4%	/	/	/	(Teng et al. 2018)
Grape	23.28%	9.82%	/	/	(Zheng et al. 2026)
Grapefruit	3.5%	36.2%	/	/	(Teng et al. 2018)
	1.152%	22.81%	3.24%	6.65%	(Wang et al. 2013)
Garlic	5.5%	52.6%	/	/	(Teng et al. 2018)
	7.19%	54.66%	2.82%	3.70%	(Teng et al. 2025)
Aloe	33.60%	0.68%	27.61%	18.62%	(Teng et al. 2025)
Lemon	1.93%	41.87%	1.08%	8.99%	(Teng et al. 2025)
Milk	/	27.6%	/	/	(Zheng et al. 2026)

Abbreviations: DGDG, digalactosyldiacylglycerol; MGDG, monogalactosyldiacylglycerol; PA, phosphatidic acid; PC, phosphatidylcholine.

A high proportion of PA appears to be a common feature of PDEVs. Owing to its small headgroup and pH‐sensitive ionization state—capable of carrying one or two negative charges depending on the environment—PA functions as a pH‐responsive lipid sensor. Its accumulation in the inner leaflet of lipid bilayers promotes negative (concave) membrane curvature, thereby facilitating membrane remodeling processes (Buratta et al. 2024). In addition, PA contributes to the gastrointestinal stability and persistence of PDEVs (Teng et al. 2018). Notably, plant‐derived miRNAs are typically methylated at the 2′‐OH of the 3′ terminal nucleotide, which confers strong resistance to degradation (Del Pozo‐Acebo et al. 2021).

After oral administration, PDEVs must traverse mucosal barriers composed of mucus and epithelium before reaching the systemic circulation (Yu et al. 2018). Their transport through mucus is governed by physicochemical properties such as particle size, zeta potential, and surface hydrophilicity (Zheng et al. 2024). EVs derived from grape and ginger have been shown to exhibit mucus penetration and subsequent cellular uptake (Zheng et al. 2026). Multiple endocytic pathways, including clathrin‐mediated endocytosis, caveolae‐mediated endocytosis, and micropinocytosis, are involved, likely due to the high lipid content of PDEVs. Notably, PA, a predominant phospholipid in plant‐derived vesicles, has been shown to facilitate transcytosis.

## 3 PDEVs in Cancer Immunotherapy: Current Advances

### 3.1 Multifaceted Roles of PDEVs in Cancer Immunity

In cancer immunotherapy, the primary objective is to reactivate the host immune system to recognize and eliminate tumor cells. Several PDEVs can intrinsically inhibit tumor cell growth and migration by inducing increased levels of reactive oxygen species (ROS) and pro‐apoptotic factors (Anusha et al. 2023; Ma et al. 2025). In addition, PDEVs enhance immunotherapeutic responses against multiple cancer types owing to their diverse repertoire of bioactive molecules.

The cancer–immunity cycle comprises several critical steps, including antigen presentation, T cell priming and activation, T cell recognition of tumor cells, and eventual T cell exhaustion. Notably, tumor cells often upregulate PD‐L1 expression to suppress T cell activity and evade immune surveillance, while also promoting an immunosuppressive tumor microenvironment (TME). PDEVs can modulate tumor–immune crosstalk through three primary mechanisms: direct immune stimulation, serving as drug delivery vehicles, and microbiota‐mediated immunomodulation (Figure 5; Table 3).

**FIGURE 5:**
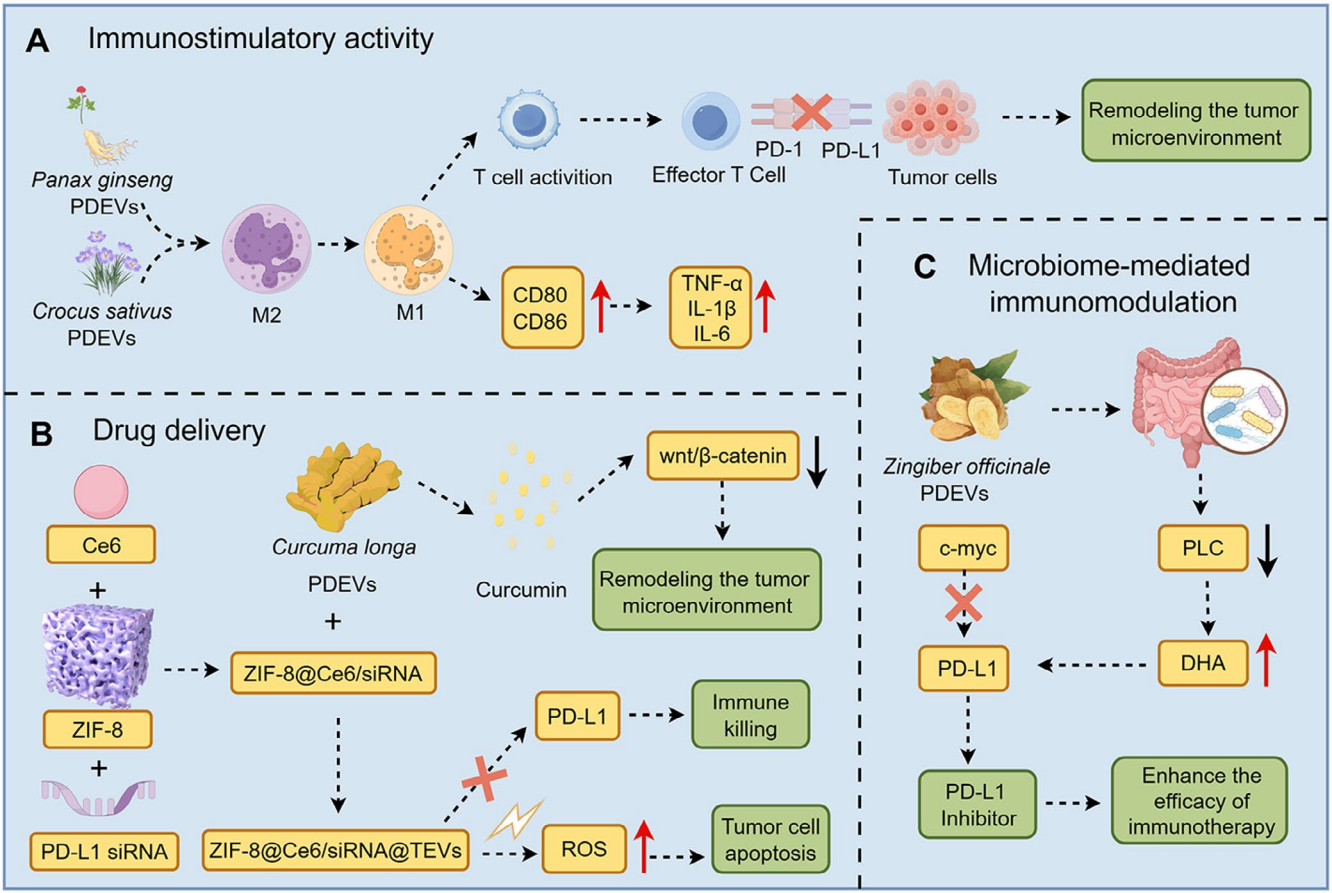
Three mechanisms of PDEVs in anti‐tumor immunity. (A) Immunostimulatory activity; (B) drug delivery; (C) microbiome‐mediated immunomodulation.

**TABLE 3:** Anti‐tumor mechanism of PDEVs.

Source	Anti‐cancer	Mechanism	Pathway	Component	Administration	Type	References
Ginger	Melanoma	Microbiome‐mediated immunomodulation	Inhibit PD‐L1 transcription in tumor cells by the gut microbiota metabolite DHA	Lipids and amino acids (PA, DGDG; glycine): uptake by Lachnospiraceae and Lactobacillaceae aly‐miR159a‐3p: target bacterial PLC leading to accumulation of DHA	Oral, combined with anti‐PD‐L1 antibody (i.p.)	Native	(Teng et al. 2025)
	Melanoma	Tumor cell suppression	Induce tumor cell apoptosis	Lipid: cytotoxic gingerols and shogaols Surface modified with tumor‐targeting ligand iRGD	i.v.	Engineered	(Wang, Li, et al. 2025)
	Triple‐negative breast cancer (TNBC)	Tumor cell suppression	Induce TNBC cell apoptosis, cell cycle arrest, and anti‐metastasis	/	In vitro	Native	(Anusha et al. 2023)
Garlic	Melanoma, colon cancer, breast cancer	Immunostimulatory activity	Promote the activation and migration of intestinal IFNγ‐producing γδ T cells into the TME, reversing its immunosuppressive state	Allicin, polyphenols, organosulfur	Oral, combined with anti‐PD‐L1 antibody (intratumoral)	Native	(Xu et al. 2024)
Ginseng	Melanoma	Immunostimulatory activity	M1‐like TAM polarization via TLR4‐MyD88 signaling	Lipids: DGMG (59.4%), PE (16.8%) and Cer; proteins	i.p.	Native	(Cao et al. 2019)
	Glioma	Immunostimulatory activity	Induce tumor cell apoptosis; M1‐like TAM polarization; blood–brain barrier (BBB) penetration	Phospholipids: PC (28.8%), TG (16.8%), and Cer (12.9%); ginsenosides; ptc‐miR396f	i.v. or intracranial	Native	(Kim et al. 2023)
	Non‐small cell lung cancer (NSCLC)	Immunostimulatory activity	Downregulate PD‐L1 expression in NSCLC cells; enhance T cell function	/	i.v., combined with anti‐PD‐L1 monoclonal antibody	Native	(Zhu et al. 2025)

	Glioma	Drug delivery	Tumor targeting and killing	Cisplatin	i.p.	Engineered	(Yang, Guo, Huang, et al. 2025)
	Cancer vaccine	Immunostimulatory activity	Promote DC maturation and T cell activation	Hybrid vesicle: ginseng‐derived nanoparticle + autologous tumor membrane	s.c. at the base of tail	Engineered	(Wang, Mu, et al. 2024)
Glioblastoma vaccine	Immunostimulatory activity	Promote DC maturation, M1‐like TAM polarization and T cell activation	Hybrid vesicle: ginseng‐derived nanoparticle (ginsenoside Rg3) + liposome (shikosin and immune adjuvant mCpG)	Intranasal	Engineered	(Wang, Zhang, et al. 2025)
Tea flower	Breast cancer	Tumor cell suppression Microbiome‐mediated immunomodulation	Induce tumor cell apoptosis	Polyphenols, flavonoids, functional proteins, and lipids	Oral or i.v.	Native	(Chen, Li, et al. 2022; Chen et al. 2023)
Celery	Lung cancer	Immunostimulatory activity Drug delivery	Inhibit the expression of PD‐L1 in lung cancer cells and activate cytotoxic T cells	Paclitaxel	i.p.	Engineered	(Lu et al. 2024)
Turmeric	Lung cancer	Immunostimulatory activity Drug delivery	Activate APCs, enhance T cell infiltration into the TME, and downregulate checkpoint PD‐L1 expression	Hybrid carrier: turmeric‐derived EVs (curcumin) + zeolitic imidazolate framework‐8; payload: photosensitizer Ce6 and PD‐L1 siRNA	i.v.	Engineered	(Yang et al. 2026)
*Artemisia annua*	Breast cancer, lung cancer	Immunostimulatory activity	M1‐like TAM polarization and T cell infiltration	mtDNA	Intratumoral; i.p. (combined with PD‐L1 antibody)	Native	(Liu et al. 2023; Wang, Meng, et al. 2025)
*Platycodon grandiflorum*	Triple‐negative breast cancer	Immunostimulatory activity Microbiome‐mediated immunomodulation	Reduce PD‐1 expression on T cells to enhance cytotoxicity M1‐like TAM polarization	Phospholipids; AGO proteins	Oral or i.v.	Native	(Yang, Guo, Li, et al. 2025)
*Pinellia ternata*	Lung cancer	Immunostimulatory activity	M1‐like TAM polarization; S‐phase cell cycle arrest in tumor cells	Lipids and proteins	Intragastric gavage	Native	(Liu et al. 2026)
*Cannabis sativa*	Multiple tumors	Immunostimulatory activity Drug delivery	Promote DC maturation, T and NK cell activation; alleviate T cell exhaustion as a vaccine delivery platform	Metabolites; ovalbumin (OVA)	Oral	Engineered	(Park et al. 2025)
Grapefruit	Melanoma	Tumor cell suppression	Induce cell cycle arrest at G2/M phase	Metabolites	In vitro	Native	(Stanly et al. 2020)
	Brain tumor, colon cancer	Drug delivery	Tumor targeting and killing	Stat3 inhibitor JSI‐124 for brain tumor; chemotherapeutic drug (paclitaxel) for colon cancer	Intranasal (brain tumor); i.v. (colon cancer)	Engineered	(Wang et al. 2013)
*Morus nigra*	Liver cancer	Tumor cell suppression Microbiome‐mediated immunomodulation	Suppress tumor cell viability, migration, and invasion	Glycolipids, proteins, and active small molecules (rutin, quercetin 3‐O‐glucoside)	Oral	Native	(Gao et al. 2024)
Brucea javanica	Triple‐Negative Breast Cancer	Tumor cell suppression	Inhibit tumor growth via PI3K‐AKT‐mTOR pathway; anti‐angiogenesis	miRNAs	Intratumoral	Native	(Yan et al. 2024)
Radix Polygoni Multiflori	Liver Cancer	Tumor cell suppression	Inhibit liver cancer cell proliferation, migration and induce cell apoptosis	/	In vitro	Native	(Yang, Xu, and Wang 2025)
*Lonicera japonica* Thunb.	HPV‐associated cervical cancer	Tumor cell suppression	Inhibit E6/E7 oncogene expression in HPV‐positive cancer cells	miR2911	In vitro	Native	(Chi et al. 2024)
Bitter melon	Oral Squamous Cell Carcinoma	Tumor cell suppression Drug delivery	Induce tumor cell apoptosis	miRNAs, and 5‐FU	peritumoral	Engineered	(Yang et al. 2021)
Asparagus cochinchinensis	Hepatocellular Carcinoma	Tumor cell suppression	Induce tumor cell apoptosis	Lipids, proteins, and RNAs; PEGylation decoration	i.v.	Engineered	(Zhang, He, et al. 2021)
*Solanum lycopersicum* (Tomato)	Colon Cancer	Drug delivery	Inhibit colon cancer cell viability and migration	Calcitriol	In vitro	Engineered	(Sarwareddy et al. 2025)
*Rhodiola rosea*	Melanoma	Drug delivery	Inhibit cell proliferation	DOX; surface modified with DSPE‐PEG2000‐pYEEIE: tumor targeting	In vitro	Engineered	(Xu, Duan, et al. 2025)
*Raphanus sativus* L. var. caudatus Alef	Colon Cancer	Tumor cell suppression	Induce cancer cell apoptosis	β‐Pleated sheet proteins	In vitro	Native	(Kaimuangpak, Rosalina, et al. 2024)
Aloe	Breast Cancer	Drug delivery	Inhibit tumor cell growth, migration, and induce cell apoptosis	ICG and DOX; tumor‐targeted RGD modification	i.v.	Engineered	(Zeng et al. 2022)
*Crocus sativus*	/	Immunostimulatory activity	Increase the expression of co‐stimulatory molecules (CD80/CD86) and pro‐inflammatory cytokines (TNF‐α, IL‐1β, and IL‐6) in macrophages	miR157, miR166, miR168, miR396, and miR398; anthocyanins	In vitro	Native	(Martínez Fajardo et al. 2025)
*Hypericum perforatum*	Melanoma	Tumor cell suppression	Photoinduced cytotoxicity against tumor cells	Hypericin	i.v.	Native	(Ma et al. 2025)
Glycyrrhiza uralensis Fisch	Melanoma	Immunostimulatory activity Drug delivery	Inhibit tumor growth; promote DC maturation; activate T cells	miR2916, isoliquiritigenin, and STING agonist DMXAA; surface‐displayed PD‐L1 antibody	i.v.	Engineered	(Xu, Yang, et al. 2025)

Abbreviations: APC, antigen‐presenting cell; Cer, ceramides; DC, dendritic cell; DGDG, digalactosyldiacylglycerol; DGMG, digalactosyl monoacylglycerol; DHA, docosahexaenoic acid; DOX, doxorubicin; Hex1Cer, hexosylceramides; i.p., intraperitoneal; i.v., intravenous; ICG, indocyanine green; PA, phosphatidic acid; PC, phosphatidylcholine; PE, phosphatidylethanolamine; PG, phosphatidylglycerol; PLC, phospholipase C; s.c., subcutaneous; TAM, tumor‐associated macrophage; TG, triglycerides; TME, tumor microenvironment.

#### 3.1.1 Tumor Cell Suppression

PDEVs derived from *Zingiber officinale* (ginger) directly induce apoptosis in triple‐negative breast cancer cells via ROS‐mediated pathways (Anusha et al. 2023). In addition, PDEVs derived from *Hypericum perforatum* (St. John's wort) act as natural photosensitizers, generating ROS upon light irradiation and inducing apoptosis in melanoma cells, accompanied by activation of the caspase‐3 and Bax signaling pathways (Ma et al. 2025). These findings highlight the cytotoxic potential of PDEVs against tumor cells.

#### 3.1.2 Immunostimulatory Activity

PDEVs can directly stimulate immune responses through distinct pathways, including: (1) antigen presentation, by promoting dendritic cell (DC) maturation and M1‐like macrophage polarization; (2) T cell activation, by enhancing cytotoxic T cell and NK cell activity; and (3) reversal of the immunosuppressive TME.

##### 3.1.2.1 Antigen Presentation

PDEVs enhance the antigen‐presenting capacity of antigen‐presenting cells (APCs). PDEVs derived from *Crocus sativus* (saffron) activate macrophages by upregulating the expression of co‐stimulatory molecules CD80 and CD86 and promoting the secretion of pro‐inflammatory cytokines such as IL‐1β, IL‐6, and TNF‐α, thereby enhancing the activation status of APCs. This effect is mainly attributed to their cargo of proteins and miRNAs (Martínez Fajardo et al. 2025).

##### 3.1.2.2 T Cell Activation

PDEVs derived from *Panax ginseng* (ginseng) alleviate the functional suppression of infiltrating CD8^+^ T cells by downregulating PD‐L1 expression on the surface of non‐small cell lung cancer cells, thereby restoring their ability to secrete TNF‐α, IL‐2, and IFN‐γ. This finding highlights the potential of PDEVs as immune checkpoint modulators, which may exert synergistic effects when combined with anti‐PD‐L1 therapy (Teng et al. 2025; Zhu et al. 2025). In addition, orally administered PDEVs derived from *Allium sativum* (garlic) induce the expansion of IFN‐γ‐producing γδ T cells in the gut, subsequently enhancing systemic anti‐tumor immune responses (Xu et al. 2024).

##### 3.1.2.3 TME Remodeling

PDEVs derived from *Platycodon grandiflorus* (balloon flower), administered orally or intravenously, promote the polarization of tumor‐associated macrophages (TAMs) toward the M1 phenotype, thereby reversing the immunosuppressive TME. This effect is associated with the induction of ROS production and the release of pro‐inflammatory cytokines, as well as modulation of the gut microbiota (Yang, Guo, Li, et al. 2025).

#### 3.1.3 Drug Delivery Vehicles

PDEVs can serve as delivery vehicles for functional cargo, including bioactive molecules and therapeutic drugs, targeting immune cells or tumor cells. *Solanum lycopersicum* (tomato)‐derived EVs have been used to encapsulate calcitriol for the treatment of colon cancer (Sarwareddy et al. 2025). *Rhodiola rosea* ‐derived EVs demonstrate targeted delivery of doxorubicin to melanoma cells (Xu, Duan, et al. 2025). *Panax ginseng* (ginseng)‐derived EVs function as drug delivery systems that reduce the required dose while enhancing the anti‐cancer efficacy of cisplatin (Yang, Guo, Huang, et al. 2025). Celery‐derived EVs act as carriers for paclitaxel and improve the efficacy of lung cancer chemotherapy by inhibiting the p‐STAT3/PD‐L1 pathway, while also promoting CD8^+^ T cell‐mediated anti‐tumor immunity (Lu et al. 2024). Turmeric‐derived EVs serve as carriers for the photosensitizer Ce6 and PD‐L1 siRNA, exerting therapeutic effects by inhibiting the Wnt/β‐catenin pathway and inducing immunogenic cell death. This, in turn, enhances immune infiltration, suppresses immune escape, and enables full‐cycle synergistic immunotherapy for lung cancer (Yang et al. 2026). *Cannabis sativa* ‐derived EVs deliver the OVA257–264 antigen and promote dendritic cell maturation via the TLR2/TLR4 signaling pathway, thereby enhancing Th1 and cytotoxic T lymphocyte (CTL) responses and demonstrating dual functionality as both an immunotherapeutic agent and a vaccine delivery platform (Park et al. 2025). Aloe‐derived EVs encapsulating indocyanine green and doxorubicin exhibit tumor‐targeting capability, inhibit cell proliferation and migration, induce apoptosis, and achieve synergistic anti‐breast cancer effects through combined photothermal and chemotherapy (Zeng et al. 2022).

#### 3.1.4 Microbiome‐Mediated Immunomodulation

Orally administered PDEVs reach the intestinal tract and modulate the composition and function of the gut microbiota, thereby indirectly regulating systemic immune homeostasis. This mechanism provides a novel framework for understanding the systemic immunomodulatory effects of PDEVs. Broccoli‐derived EVs regulate the gut microbiota–associated metabolite sulforaphane, activate the adenosine monophosphate–activated protein kinase (AMPK) signaling pathway in dendritic cells, and induce tolerogenic dendritic cells, thereby preventing colitis in mice (Deng et al. 2017). Tea leaf‐derived EVs modulate the gut microbiota while also activating ROS‐mediated mitochondrial damage and cell cycle arrest pathways, leading to apoptosis of breast cancer cells and inhibition of tumor growth (Chen et al. 2023). Ginger‐derived nanoparticles transport encapsulated aly‐miR159a‐3p to gut bacteria, where it suppresses phospholipase C (PLC) expression, resulting in increased accumulation of docosahexaenoic acid (DHA). DHA then interacts with the PD‐L1 promoter in tumor cells, suppresses PD‐L1 transcription, and enhances the therapeutic efficacy of anti‐PD‐L1 immunotherapy against melanoma (Teng et al. 2025). In these cases, the biological effects of PDEVs are mainly mediated through modulation of the intestinal microecology rather than direct action on tumor or immune cells.

### 3.2 PDEVs in Cancer Immunotherapy: Clinical Translation

Despite substantial growth in preclinical research on PDEVs, their clinical translation remains at an early stage and is largely confined to initial clinical investigations. A limited number of registered studies—such as those evaluating engineered grape‐derived exosomes for drug delivery in colon cancer (NCT01294072), native PDEVs for the treatment of inflammatory bowel disease (IBD) and colitis‐associated cancer (NCT04879810), and for the prevention of oral mucositis in head and neck cancer (NCT01668849)—represent early steps toward clinical application. An overview of these pioneering PDEV‐based interventions currently under clinical investigation is presented in Table 4 (data obtained from ClinicalTrials.gov).

**TABLE 4:** Registered clinical trials of oral PDEVs.

No.	Source	Disease	Stage	Start year and location	Clinical trial ID
1	Grape	Colon cancer	Phase I	2011, United States	NCT01294072
2	Ginger	IBD and colitis‐associated cancer	Pilot	2018, United States	NCT04879810
3	Grape	Oral mucositis associated with chemoradiation treatment of head and neck cancer	Phase I	2012, United States	NCT01668849

## 4 Challenges and Prospects in the Clinical Application of PDEVs

Despite their considerable potential for biomedical applications, PDEVs still face significant translational challenges. At the same time, emerging engineering strategies and the integrative theoretical framework of TCM have opened promising avenues for future development in this field.

### 4.1 Challenges

#### 4.1.1 Current Challenges and Critical Barriers in PDEV Research

##### 4.1.1.1 Heterogeneity and Lack of Standardization in PDEV Preparations

Despite growing interest in PDEVs for biomedical applications, challenges related to vesicle heterogeneity and the lack of standardized protocols remain insufficiently addressed (Lian et al. 2022). PDEVs exhibit substantial intrinsic variability arising from plant species, tissue sources (e.g., roots, leaves, fruits), cultivation conditions (e.g., climate, soil) (Park et al. 2025), and post‐harvest processing (Ferroni and Zavan 2025). These factors collectively influence vesicle composition, size distribution, and functional properties, leading to batch‐to‐batch inconsistencies that compromise reproducibility and cross‐study comparability. Unlike mammalian exosomes, which have well‐established markers (e.g., CD9, CD63, CD81), PDEVs lack universally validated biomarkers. Although candidate proteins such as PEN1 and TET8 have been proposed, their species‐ and tissue‐dependent expression may limit their generalizability as reliable purification markers (Ghosh and Innes 2025; Koch et al. 2025). The application of IAC is limited due to the absence of standardized markers and the scarcity of specific antibodies used to identify different plants. To date, TET8‐positive EVs have been isolated from *Arabidopsis thaliana* apoplastic fluid using antibody‐conjugated beads targeting specific domains of TET8 (He et al. 2021; Huang et al. 2021). Furthermore, although three PDEV biogenesis routes—MVBs, EXPO, and vacuoles—have been proposed, the relative quantitative contribution of each pathway across diverse plant species remains poorly characterized. Key unresolved questions include whether TET8‐positive vesicles identified in *Arabidopsis thaliana* are conserved across the plant kingdom, and whether EXPO pathway activation thresholds exhibit species‐specific regulation under different stress conditions (Song et al. 2025).

Current challenges also include scalable manufacturing under good manufacturing practice (GMP)–compliant conditions, the establishment of standardized operating procedures, and a clearer mechanistic understanding of cargo loading into PDEVs. Addressing these issues will be essential for advancing PDEVs as drug delivery systems and functional food ingredients.

Based on current evidence, a three‐tiered purification workflow has been proposed to address challenges of heterogeneity and scalability in PDEV research:

Primary workflow (basic research grade): differential ultracentrifugation (DUC) coupled with size‐exclusion chromatography (SEC). The combination of DUC and SEC significantly enhances purity, as SEC can efficiently remove more than 95% of co‐purifying soluble protein contaminants (Bokka et al. 2020).

Secondary workflow (functional studies grade): DUC combined with high‐resolution density gradient ultracentrifugation and SEC. This approach enables separation of distinct vesicle subpopulations (e.g., TET8^+^ vs. PEN1^+^ populations) (Koch et al. 2025), achieving the highest purity. It is particularly suitable for subpopulation functional analysis and marker identification.

Tertiary workflow (production grade): tangential flow filtration integrated with anion exchange chromatography and SEC. This strategy offers automated, continuous processing suitable for large‐scale manufacturing and achieves 3–5‐fold higher recovery rates compared with ultracentrifugation‐based methods (Giancaterino and Boi 2023; Zanotti et al. 2025; Zeng et al. 2024).

##### 4.1.1.2 Knowledge Gaps in PDEV Pharmacokinetics

In contrast to mammalian exosomes, the absorption, distribution, metabolism, and excretion profiles of PDEVs in mammalian systems remain largely unexplored. Fundamental questions remain regarding how different routes of administration (oral, intravenous, transdermal) influence biodistribution (Wang et al. 2013), whether these vesicles exhibit organ‐specific accumulation, and the precise mechanisms by which their unique lipid composition contributes to in vivo stability. To date, research on PDEVs remains predominantly preclinical, with clinical evaluation limited to a small number of Phase 1 trials (Feng et al. 2023).

#### 4.1.2 Controversies in PDEV Research

##### 4.1.2.1 Inconsistent Nomenclature Across Studies

Owing to the heterogeneity of PDEVs arising from variations in plant sources and isolation methodologies (e.g., natural secretion, mechanical disruption via tissue homogenization, and thermal extraction), diverse terminologies have been used in the literature, including “extracellular vesicles” (Yang, Guo, Li, et al. 2025), “exosomes” (Zhu et al. 2025), “extracellular vesicle‐like nanoparticles” (Wang, Mu, et al. 2024), “exosome‐like nanoparticles” (Kim et al. 2023), “nanoparticles” (Teng et al. 2025), “exosome‐like nanovesicles” (Chen, Li, et al. 2022). Recently, heat‐stable decoctosomes (exosome‐like nanoparticles) isolated from herbal decoctions have been shown to mediate small RNA absorption following oral administration (Li et al. 2019).

##### 4.1.2.2 Insufficiently Characterized Molecular Mechanism

The molecular basis of cross‐kingdom regulation by plant‐derived miRNAs remains unclear. Key questions include: (i) how dietary plant miRNAs resist degradation in the mammalian digestive tract; (ii) the mechanisms governing their uptake by host or microbial cells; and (iii) the functional consequences of this uptake at the molecular, cellular, and physiological levels.

Some studies suggest that dietary plant miRNAs are absorbed in the stomach in an SIDT1‐dependent manner (Chen et al. 2021), whereas others report that they are encapsulated within EVs and internalized in the gut either by intestinal macrophages (Wang et al. 2014) or by gut bacteria (Teng et al. 2018). Notably, PDEV surface lipids and amino acids may act as “eat me” or “don't eat me” signals for human gut bacteria (Teng et al. 2025). These divergent uptake routes likely reflect the molecular form of plant miRNAs, whether they exist as free molecules, protein‐bound complexes, or cargo within EVs. Consequently, dietary plant miRNAs may contribute to human health through two distinct pathways: (i) direct post‐transcriptional regulation of host gene expression (Hou et al. 2018; Tian et al. 2016; Zhang et al. 2012) via RNA interference mechanisms; and (ii) local modulation of gut microbial composition and function (Li, Ding, et al. 2024; Teng et al. 2025, 2018; Wang et al. 2014). These two pathways are not mutually exclusive; rather, they may operate concurrently and in a coordinated manner to mediate the biological effects of dietary plant miRNAs. Moreover, certain plant miRNAs can directly target viral or bacterial transcripts. For example, honeysuckle‐encoded miR2911 has been reported to inhibit influenza A virus replication (Zhou et al. 2015), while ginger‐derived rlcv‐miR‐rL1‐28‐3p targets SARS‐CoV‐2 RNA (Teng et al. 2021). While these findings provide compelling evidence for cross‐kingdom regulatory activity, they also raise critical questions: does the observed target complementarity reflect evolutionary coincidence, or does it indicate a broader, underappreciated regulatory potential within plant miRNA repertoires? At the same time, the extent to which dietary plant miRNAs regulate mammalian gene expression remains debated, particularly with respect to their stability during gastrointestinal transit, systemic bioavailability, dose–response relationships, and ability to be incorporated into endogenous RISCs in mammalian cells.

Collectively, research on cross‐kingdom miRNA effects remains at an early stage. To date, only a limited number of plant miRNAs, including miR168a, miR156a, and miR2911 (Lei et al. 2026), have been extensively studied. A systematic investigation of miRNAs from a broader range of edible and medicinal plants is needed to elucidate their uptake kinetics and physiological functions. Importantly, strategies for harnessing plant miRNAs for clinical applications—including delivery optimization, pharmacokinetic profiling, and safety evaluation—should be proactively incorporated into future research directions.

### 4.2 Prospects

#### 4.2.1 Engineering of PDEVs

To address the aforementioned challenges and fully realize the therapeutic potential of PDEVs, future research may build upon established engineering strategies across four key dimensions.

First, surface functionalization: chemical modification strategies, such as click chemistry, can be used to covalently couple targeting ligands to the surface of extracted PDEVs, offering a promising approach to enhance targeting specificity. For example, grapefruit‐derived PDEVs have been functionalized with aptamers via click chemistry by introducing a maleimide‐containing lipid moiety, resulting in significantly enhanced selective uptake by brain cells (hCMEC/D3 and U87MG) (Moon et al. 2023). This approach enables the development of low‐cost, scalable, targeted drug delivery systems. In addition, cRGD peptide‐modified EVs derived from lemons and oranges have been used to target tumor αvβ3 integrin for the delivery of doxorubicin (Long et al. 2023; Xiao et al. 2022). Grapefruit‐derived EVs modified with an aptamer enable targeted delivery to HER2‐positive breast cancer cells (Tang et al. 2020). Modification of asparagus‐derived EVs with PEG extends their circulation half‐life (Zhang, He, et al. 2021). In addition, PDEVs conjugated with folic acid can target tumor cells that overexpress folate receptors (Zhang, Xiao, et al. 2016; Zhuang et al. 2016).

Second, cargo loading: Techniques such as electroporation, sonication, and freeze–thaw cycles have been successfully adapted to load exogenous therapeutic molecules into the lumen of PDEVs. Comparative studies evaluating small interfering RNA (siRNA) loading efficiencies in tobacco‐derived PDEVs using methods including sonication, saponin‐assisted incubation, lipofection, and electroporation (Kocholatá et al. 2024) demonstrate the feasibility of efficient nucleic acid encapsulation while preserving vesicle integrity. Doxorubicin‐loaded ginger‐derived EVs have shown therapeutic efficacy against colon cancer (Zhang, Xiao, et al. 2016). Watermelon‐derived EVs encapsulating miR‐146a‐5p exert therapeutic effects against ovarian cancer (Corvigno et al. 2024). Grapefruit‐derived EVs loaded with siRNA via microfluidic fabrication enable effective gene silencing (Itakura et al. 2023). Orange‐derived EVs encapsulating SARS‐CoV‐2 mRNA were administered via oral or nasal routes to induce immune responses (Pomatto et al. 2023). Grapefruit‐derived EVs encapsulating bovine serum albumin and heat shock protein 70 (HSP70) exhibited cytoprotective effects (Garaeva et al. 2021).

Third, controlled release: The integration of stimuli‐responsive materials sensitive to pH, enzymes, temperature, or light with PDEVs represents an emerging strategy for achieving spatiotemporally controlled drug release at pathological sites (Lu et al. 2025). Recent studies suggest that engineered PDEVs responsive to TME cues, such as low pH or specific enzymes, as well as external physical stimuli, including light and ultrasound, hold promise for synergistic therapeutic effects and advanced targeted delivery platforms, although most of these approaches remain at the conceptual or early experimental stage for PDEVs (Tao et al. 2026).

Fourth, hybrid vesicles: Membrane hybridization or coating of PDEVs with heterogeneous cell membranes confers multifunctional biological properties, enabling enhanced targeting specificity and immune modulation. For example, hybrid bacterial–plant vesicles produced by fusing spinach‐derived EVs with *E. coli* outer membrane vesicles have been shown to enhance anti‐tumor immune responses (Zhuang et al. 2022). Similarly, hybridization of ginseng‐derived EVs with autologous tumor cell membranes effectively promotes the activation of tumor‐specific CTLs (Wang, Mu, et al. 2024). In addition, neutrophil membrane–camouflaged ginseng‐derived EVs demonstrate targeted delivery to inflamed lung tissue and ameliorate sepsis‐related symptoms (Ma et al. 2024). Likewise, activated T cell membrane‐coated grapefruit‐derived EVs exhibit efficient targeting of inflamed tumor sites (Wang et al. 2015).

Based on the engineering strategies of PDEVs discussed above, a stage‐specific translational framework is proposed. In the research and development stage, chemical modification provides a cost‐effective and scalable approach, making it well‐suited for target validation and preclinical investigations (Moon et al. 2023). In the clinical translation stage, the use of integrated purification and modification platforms is recommended to minimize intermediate processing steps and reduce storage‐related costs (Zanotti et al. 2025). For long‐term industrial production, genetic engineering approaches offer a sustainable “one‐time engineering, continuous production” model, with the potential to substantially reduce marginal costs associated with large‐scale manufacturing over extended periods.

#### 4.2.2 PDEVs and TCM

PDEVs provide unprecedented molecular tools for investigating the scientific basis of TCM theories (Wang, He, et al. 2025).

Investigating the biological effects of PDEVs isolated from TCM herbs with contrasting thermal properties (e.g., “cold” vs. “hot”) on host energy metabolism and immune regulation offers a promising approach to validating TCM classification systems at the cellular and molecular levels (Li et al. 2022). Given that PDEVs derived from herbs with distinct properties exhibit differences in lipid composition and secondary metabolites, it is hypothesized that these molecular signatures may reflect the “cold” and “hot” attributes. Emerging evidence further suggests that such property‐associated differences are reflected in their divergent modulation of gut microbiota (Li et al. 2022) and host metabolic profiles (Wang et al. 2023; Yang, Wu, Wei, et al. 2025). Specifically, hot‐natured TCMs increased α‐diversity and reduced the Firmicutes‐to‐Bacteroidetes (F/B) ratio, whereas cold‐natured TCMs exhibited a more pronounced effect, inducing substantial restructuring of the gut microbial community (Zhang, Yang, et al. 2021). Moreover, these microbiota alterations were associated with distinct serum metabolic shifts: short‐term intervention with cold‐natured TCMs reduced lactic acid levels, while long‐term administration increased tryptophan and decreased proline and threonine concentrations. Notably, hot‐natured herbs (e.g., *Astragalus membranaceus*, *Angelica sinensis*, and *Panax ginseng* ) increased metabolic‐modulating taxa such as Clostridia and *Eubacterium coprostanoligenes* , whereas cold‐natured herbs (e.g., *Rheum palmatum* , *Scutellaria baicalensis*, *Senna alexandrina* , and *Coptis chinensis*) promoted the growth of anti‐inflammatory genera such as Akkermansia and Bifidobacterium (Yang, Shao, Liu, et al. 2025). These coordinated microbiota–host interactions provide mechanistic support for the classical TCM concept of “property‐specific actions.” To evaluate whether PDEVs themselves function as carriers of herbal thermal properties, we characterized PDEVs derived from representative cold‐ and hot‐natured herbs (Table 5), focusing on their lipid composition, biodistribution, and effects on the gut microbiota. PDEVs are selectively taken up by gut bacteria, a process influenced by multiple factors, including plant source, PDEV subtype, and the intestinal microenvironment. Specific lipid and amino acid components on the vesicle surface may function as “eat me” or “don't eat me” signals; for example, DGDG and glycine promote the uptake of ginger‐derived EVs by *Lachnospiraceae* and *Lactobacillaceae*, whereas phosphatidylinositol (PI) and valine in aloe‐ and ginger‐derived EVs, respectively, inhibit bacterial uptake (Teng et al. 2025).

**TABLE 5:** TCM theory‐informed PDEV profiling.

Source	Property	Lipid	Distribution	Gut microbiota	References
Ginger	Hot	PA, DGDG	Gut	In vivo: uptake by Lactobacillusaceae (MGDG, glycine‐induced), Bacteroidales S24‐7, Clostridium (DGDG‐induced); NOT by Ruminococcus (valine‐inhibited) In vitro culture: Lactobacillus ↑; Ruminococcaceae ↓	(Teng et al. 2025, 2018)
Turmeric	Hot	PA	/	In vivo: uptake by Lactobacillusaceae, Bacteroidales S24‐7	(Teng et al. 2018)
Garlic	Hot	PC, PE	/	In vivo: uptake by Ruminococcaceae, Bacteroidales S24‐7	(Teng et al. 2025, 2018)
Grapefruit	Cold	PC	Liver, spleen	In vivo: uptake by Ruminococcaceae, Bacteroidales S24‐7 In vitro culture: Lactobacillus ↓	(Teng et al. 2018; Wang et al. 2013)
lemon	Cold	PC, PE	/	In vivo: uptake by Lachnospiraceae, Bacteroidaceae	(Teng et al. 2025)
Aloe	Cold	PA, PI	/	In vivo: uptake by Lachnospiraceae, Bacteroidaceae; NOT by Lactobacillaceae (PI‐inhibited)	(Teng et al. 2025)

Abbreviations: DGDG, digalactosyldiacylglycerol; MGDG, monogalactosyldiacylglycerol; PA, phosphatidic acid; PC, phosphatidylcholine; PE, phosphatidylethanolamine; PI, phosphatidylinositol.

Systematic in vivo biodistribution mapping of PDEVs derived from diverse herbal sources may provide a physiologically tractable correlate to the TCM concept of meridian affinity (e.g., vesicles from cough‐ and asthma‐relieving herbs can accumulate in lung tissue) (Fu et al. 2025). PA‐enriched PDEVs prolong intestinal retention, whereas PC‐dominant PDEVs facilitate transintestinal migration to the liver (Teng et al. 2018). Furthermore, ginger‐derived EVs induce DHA/EPA production specifically in the small intestine, suggesting site‐specific bioactivity along the gastrointestinal tract (Teng et al. 2025).

In the context of compound formulations, integrative analysis of how PDEVs from multiple herbal sources cooperatively engage distinct targets and pathways—driven by complementary cargo profiles—offers a modern interpretation of the classical “sovereign, minister, assistant, and courier” formulation principle (Li et al. 2017). Recent studies have also identified naturally self‐assembled nanoparticles in TCM decoctions (Fang et al. 2025; Huang et al. 2023; Lin et al. 2022).

To advance mechanistic validation of the Cold‐Hot theory at the PDEV level, future studies should expand herb representation across the full thermal spectrum, rigorously dissect the relationships among herbal nature, biodistribution, and biological function, and implement a phased multi‐omics roadmap: (i) establishment of a standardized, annotated herb panel stratified by validated thermal properties; (ii) comprehensive molecular phenotyping, including quantitative lipidomics (LC–MS/MS), metabolomics, surface proteomics, and small RNA sequencing; and (iii) integrated functional validation in physiologically relevant models (e.g., mice), linking PDEV molecular features to thermal phenotypes and target engagement.

## 5 Conclusion

PDEVs derived from edible and medicinal plants represent a versatile and promising platform for cancer immunotherapy. Their ability to inhibit tumor cells, stimulate anti‐tumor immunity, deliver therapeutic agents, and modulate gut microbiota underscores their multifunctional potential. Compared with mammalian exosomes, PDEVs offer advantages in cost‐effectiveness, scalability, and gastrointestinal stability, supporting their development as natural drug delivery systems and functional food components. Nevertheless, clinical translation of PDEVs remains in an early stage. Major challenges include heterogeneity across plant sources, lack of standardized isolation and characterization protocols, and limited understanding of pharmacokinetics. Addressing these issues will require the establishment of validated biomarkers, scalable GMP‐compliant production methods, and rigorous in vivo functional studies. Future research should focus on engineering strategies to enhance targeting, stability, and controlled release. By bridging TCM theories with modern nanotechnology, PDEVs offer a unique lens for mechanistic exploration of dietary and herbal interventions.

## Author Contributions

**Guosheng Li:** conceptualization, resources, investigation, writing – original draft, writing – review and editing. **Mengyu Li:** investigation. **Shujing Liu:** investigation. **Yuan Wei:** supervision, validation. **Chen Wang:** conceptualization, resources, investigation, supervision, validation, writing – original draft, writing – review and editing.

## Funding

This work was supported by Senior Talent Foundation of Jiangsu University (5501290013 to Chen Wang).

## Conflicts of Interest

The authors declare no conflicts of interest.

## Data Availability

This review does not contain any original data or code. All data and resources referenced in this article were obtained from previously published studies, which are cited accordingly.
